# Study of Polyhedral Oligomeric Silsesquioxane-Modified Superhydrophilic Transparent Coating in Antifogging, Antifrost and Self-Cleaning

**DOI:** 10.3390/polym17050599

**Published:** 2025-02-24

**Authors:** Weibiao Zhu, Chengfeng Wu, Jinxin He, Xia Dong

**Affiliations:** 1 College of Chemistry and Chemical Engineering, Donghua University, Shanghai 201620, China; shhy_zwb@163.com (W.Z.); wu.chengfeng@huafeng.com (C.W.); dongxia@dhu.edu.cn (X.D.); 2 Shanghai Hangyong Photoelectric New Materials Co., Ltd., Shanghai 201600, China

**Keywords:** superhydrophilic, antifogging, antifrost, self-cleaning

## Abstract

A highly hydrophilic coating with a water contact angle below 10° was developed on a transparent polycarbonate (PC) sheet using a UV-curable process. Polyhedral oligomeric silsesquioxanes (POSS), modified with poly(ethylene glycol) methacrylate (PEGMA), provided the hydrophilic functionality essential for the coating. Fogging and frost formation on transparent surfaces often hinder visibility and pose challenges in various optical applications. The hydrophilic coating developed in this study demonstrated excellent antifogging and antifrost properties, along with a notable self-cleaning capability. These characteristics make it a promising candidate for applications in transparent optical PC materials, requiring enhanced antifogging, antifrost, and self-cleaning performance.

## 1. Introduction

Transparent materials play a crucial role in daily life, with applications in goggles, spectacle lenses, windshields, dental mirrors, plastic packaging, and endoscope lenses [1]. However, their transparency is often compromised by ambient temperature and humidity, leading to fogging or frosting. When the surface temperature of a transparent material falls below the dew or frost point of ambient water vapor, uneven condensation of water droplets occurs [2]. These tiny, irregularly distributed droplets scatter and refract visible light, resulting in a hazy appearance and significantly reduced transparency [3]. Fogging and frosting not only cause inconvenience but can also pose serious challenges. For instance, in food packaging, condensed water droplets affect both esthetics and food quality. In agricultural films, reduced transparency due to fogging can inhibit crop growth and damage plant leaves. In occupational settings where goggles are essential for protection against exposure and infection, persistent fog formation can hinder visibility, making it difficult for users to wear them for extended periods, especially during precision procedures [4,5]. While controlling ambient temperature and humidity is the most straightforward way to prevent fogging or frosting, this approach is energy-intensive, costly, and environmentally unsustainable [6]. As a result, antifogging and antifrost materials that prevent surface water condensation have garnered significant research interest and are now considered essential for many optical applications [7,8].

Surface modifications have been reported to have good antifogging and antifrost behavior. Zheng et al. developed dual-functional antifogging/antifrosting coatings with organic–inorganic zwitterionic polymer via free radical copolymerization using quaternary ammonium salt as a hydrophilic monomer [9]. Manabe et al. developed a surface coating composited with graphene within a polymeric multilayer film exhibiting simultaneous antifogging and frost-resist properties [10]. Yang et al. fabricated a multifunctional coating with good antifog and antifrost properties on a glass plate using butyl acrylate (BA), acrylic acid (AA), and 4-benzoylphenyl acrylate (BPA) as the polymer monomers [11]. In addition, micro/nano-composite structures which effectively prevent condensed water droplets from rapidly spreading on their surface can be applied in antifogging and antifrost materials [12].

Most research on antifogging and antifrost coatings has focused on two primary mechanisms: superhydrophobicity (water contact angle > 150°) and superhydrophilicity (water contact angle < 5°) [1]. In some cases, antifogging effects can also be achieved on hydrophilic surfaces with contact angles below 40° [8]. Superhydrophobic surfaces prevent water droplets from adhering by causing them to roll off, thereby minimizing random light scattering and preserving transparency. However, their fabrication is often complex, requiring rough surface structures that can reduce transparency [8]. Additionally, superhydrophobic surfaces are prone to fouling, limiting their long-term performance [1]. In contrast, superhydrophilic surfaces allow water droplets to spread evenly, forming a continuous film that prevents light scattering and maintains high transparency [1]. Beyond antifogging and antifrost properties, hydrophilicity also plays a crucial role in self-cleaning, making it essential for optical applications that require high transparency and efficiency. A key challenge, however, lies in developing scalable manufacturing processes for large transparent materials. The UV-curable method is widely used in continuous fabrication due to its ability to produce coatings efficiently [13]. This approach minimizes the use of volatile organic compounds, reducing environmental impact while enhancing polymerization rates and production efficiency [14,15].

In related research, hydrophobic components have been shown to regulate the dispersion of water molecules in coatings [16]. Polyhedral oligomeric silsesquioxanes (POSS) consist of an inorganic silica core surrounded by organic functional groups (R), which facilitate polymerization or grafting reactions [17]. Introducing compounds containing polyether segments into POSS reduces the agglomeration effect of nano-POSS on one hand, and increases the compatibility of POSS with polyethylene glycol acrylate resins on the other hand. Modified POSS can be dispersed at the nanoscale within the coating, thereby not affecting the transparency of the coating. Previously, we developed a superhydrophobic coating containing POSS with high transparency [18].

There were various methods to prepare the super-wetting surface, but many methods require high-temperature calcination or baking curing, which was not suitable for temperature-sensitive transparent plastic substrate, and most of the special surface-wetting treatment was expensive, time-consuming, and high energy consumption, it is difficult to achieve large area and rapid preparation process. Different from many researchers on the preparation of performance surfaces such as anti-fogging and antifrost protection of materials [19,20,21,22], we were committed to introducing the current large-area industrial coating method to the special wetting surface while making the radiation-curing raw materials at low temperatures, so that the temperature-sensitive plastic substrate can also undergo the large-area, low-temperature, fast-curing process to obtain transparent super wetting coating (A list of differences from similar studies was described in Supplementary File S1). In this study, we aimed to enhance hydrophilicity by optimizing the POSS network structure through the introduction of ethylene glycol groups. The grafted ethylene glycol groups are expected to create synergistic effects, promoting water molecule dispersion, and improving the coating’s performance.

## 2. Materials and Methods

### 2.1. Materials

All chemicals were used as received without any further purification. Polycarbonate (PC) with an original dimension size of 2 cm × 10 cm × 0.8 mm was purchased from Sichuan Longhua Photoelectric Film Co., Ltd. (Mianyang, China) and used as substrate material. 2-methyl-4-methyl thio-2-morpholine phenylacetone (2-M-4-MT-2-MP, product name I-907, 98%) was purchased from Shanghai Aladin Biochemical Technology Co., Ltd. (Shanghai, China). Anhydrous tetrahydrofuran (THF, 98%), anhydrous ether and anhydrous ethyl alcohol (EtOH, 99.5%) were purchased from Sinopharm Chemical Reagents Co., Ltd. (Shanghai, China). 1,6-Hexanediol diacrylate (HDDA) was purchased from Eternal Chemical Co., Ltd. (Suzhou, China). POSS (molecular weight 633.04, purity 98%) was purchased from Suzhou Xisuo Co., Ltd. (Suzhou, China). 1,2-Dimercaptoethane was purchased from Shanghai Titan Technology Co., Ltd. (Shanghai, China). Poly(ethylene glycol) dimethacrylate (PEGDMA, molecular weight 600) was purchased from Shanghai Aladin Biochemical Technology Co., Ltd. (Shanghai, China). Water used throughout coating preparation and characterization was deionized. POSS-SH_8_ was synthesized and purified independently [23].

### 2.2. Preparation Process

#### 2.2.1. Preparation of POSS-SH_4_-EG_4_

PEGMA (8.237 g, 0.01735 mol) was dissolved in 20 mL of THF, designated as solution C. POSS-SH_8_ (6.00 g, 0.00446 mol) was weighed and dispersed in 15 mL of THF, followed by the addition of I-907 (0.06 g), forming solution D. Solution C was gradually added to solution D at a rate of 10 mL/h. After the addition was complete, the reaction mixture was stirred for an additional 30 min. The resulting mixture was then filtered through a polytetrafluoroethylene (PTFE) membrane to remove insoluble substances. Partial solvent removal was performed using rotary evaporation at 35 °C. The remaining solution was transferred to a centrifuge tube and centrifuged at 8000 r/min for 10 min using EtOH as the precipitating agent. A white waxy substance settled at the bottom, the target product remained in the intermediate layer, and THF formed the supernatant. The target product was carefully extracted and washed 5–8 times with anhydrous ether to remove residual I-907. Finally, anhydrous ether was evaporated under vacuum at 30 °C for 24 h, yielding a faint yellow viscous product, designated as POSS-SH_4_-EG_4_. Modified POSS-SH8 with different substitution numbers were synthesized, such as POSS-SH_6_-PEGMA_2_, POSS-SH_4_-PEGMA_4,_ and POSS-SH_2_-PEGMA_6_. Considering the POSS content, transparency, and appearance of the coating, POSS-SH_4_-PEGMA_4_ was selected as the best coating component among the above-mentioned components. In addition, the unmodified thiol group can participate in the curing of the coating to reduce the loss during application.

#### 2.2.2. Preparation of the Coating

The protective films on both sides of the substrate (PC) with the dimension size of 2 cm × 10 cm were turned off gently with tweezers. Then, the substrate was cleaned by EtOH to remove the contaminants on the surface and rinsed with deionized water, then dried with soft cotton textile gently.

The synthesized POSS-SH_4_-EG_4_ in clause 2.2.2 and PEGDMA were mixed by the weight ratio of 7:3, named mixture I. Then, I-907 (1 wt%) and hexanediol diacrylate (HDDA, 2 wt%) were added to the mixture I and mixed evenly to form mixture II. After mixing, the coating was prepared by a scraper coating machine onto the surface of the PC. Then, the coating was cured for 30 S under a 3000 W mercury lamp with a curing energy of approximately 1000 MJ/cm^2^. Without POSS-SH_4_-EG_4_, the mixture containing PEGDMA, I-907, and HDDA (wt% = 97:1:2) was named N-coating and the corresponding PC was named N-coated PC. The ethoxy group in PEGDMA mainly provided hydrophilicity for the coating. HDDA provided adhesion for the coating and reduced the component viscosity to facilitate the coating construction.

### 2.3. Characterization

The chemical structures of POSS-SH_8_ and POSS-SH_4_-EG_4_ were characterized using Fourier transform infrared (FTIR) spectroscopy and proton nuclear magnetic resonance (^1^H NMR) spectroscopy.

#### 2.3.1. FTIR Spectroscopy

FTIR spectroscopy analysis was carried out by means of a Bruker spectrometer (TENSOR 27, Bruker GmbH, Karlsruhe, Germany) interferometer. Measurements were conducted between 450 and 4000 cm^−1^, with a 4 cm^−1^ resolution. The spectra were taken in the attenuated total reflection mode.

#### 2.3.2. H NMR Spectroscopy

Nuclear magnetic resonance hydrogen spectrum (1H NMR) was conducted with a Bruker spectrometer (AV400 M, Bruker GmbH, Karlsruhe, Germany). The samples were dissolved in CDCl3 resolution with a concentration of 10–20 mg/mL. Tetramethylsilane (TMS) was used as the internal standard to record the resonance signal spectrum of hydrogen atoms.

#### 2.3.3. UV-Visible Near-Infrared Spectroscopy

The transmittance of the samples was measured using a UV-visible spectrophotometer (UV3200, Shanghai Mepuda Instrument Co., Ltd., Shanghai, China.) with a test range of 300–800 nm.

#### 2.3.4. X-Ray Photoelectron Spectroscopy (XPS)

X-ray photoelectron spectroscopy was used to make elements on the surface of the coating. The XPS test range was a circle with a diameter of 100 μm.

#### 2.3.5. Scanning Electron Microscopy (SEM)

The surface of the coating sample was treated with gold spray, and the surface morphology was observed by scanning electron microscope (SEM) under the accelerated voltage of 15 kV.

#### 2.3.6. Surface Wettability Assessment

The hydrophilic behavior of the coating was evaluated by measuring the water contact angle (WCA) using a contact angle measurement system (DSA30, KRÜSS GmbH, Darmstadt, Germany). Measurements were performed at room temperature using deionized water. A droplet volume of 5 μL was consistently maintained, and the droplet was dispensed at a controlled rate of 2 μL/s. For each sample, measurements were taken at five different locations to ensure accuracy, and the average WCA value was reported with an estimated maximum error of ±3°. The contact angle was determined based on the recorded images of dispensed and static water droplets on the coated surface, providing a quantitative measure of surface wettability.

#### 2.3.7. Antifogging Performance Evaluation

The antifogging capability of the coating was assessed by exposing both coated and uncoated polycarbonate (PC) samples to hot vapor. The test was conducted using two different water temperatures: 100 °C and 50 °C. A beaker containing 100 mL of heated water was used to generate hot vapor, and both the uncoated blank PC and the coated PC samples were simultaneously placed above the beaker. A written paper was positioned beneath the setup to serve as a visual reference for transparency assessment. After 15 s of exposure to hot vapor, the extent of fogging on the surfaces was observed and recorded using a mobile camera. The comparison of water droplet condensation patterns on both surfaces provided qualitative evidence of the coating’s antifogging performance.

#### 2.3.8. Antifrost Performance Evaluation

To evaluate antifrost properties, both coated and uncoated PC samples were subjected to freezing conditions. The samples were stored in a freezer at −5 °C for 15 min to allow frost formation. After freezing, they were exposed to ambient conditions, and a written paper was placed beneath the samples to assess haze formation. Any differences in frost accumulation and transparency retention were visually documented using a mobile camera. This experiment demonstrated the coating’s ability to inhibit frost formation and maintain optical clarity.

#### 2.3.9. Self-Cleaning Performance Evaluation

The self-cleaning properties of the coating were tested using peanut oil dyed red as a model contaminant. Droplets of the dyed oil were applied to the surfaces of both the coated and uncoated PC samples. The behavior of the oil droplets on the surfaces was observed and recorded using a mobile camera. The ease with which the oil droplets spread, rolled off, or remained adhered to the surface was used as an indicator of the coating’s self-cleaning efficiency.

## 3. Results and Discussion

### 3.1. Characterization of POSS-SH_8_ and POSS-SH_4_-EG_4_

**Figure 1:**
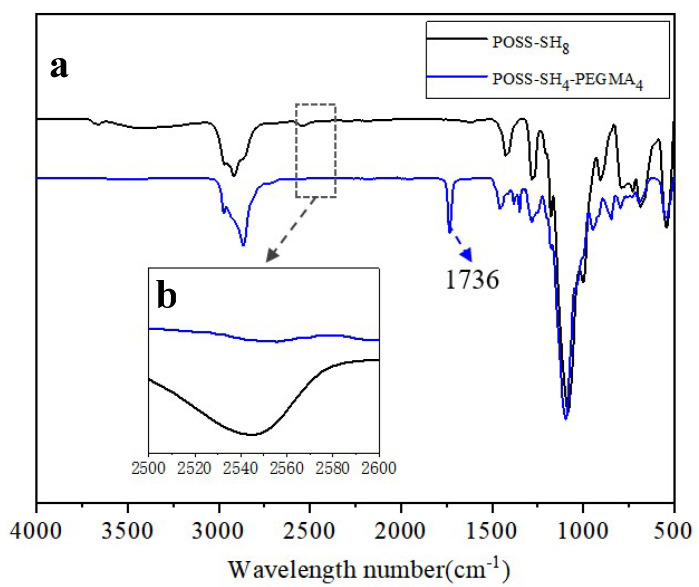
Typical FTIR spectra of POSS-SH_8_ and POSS-SH_4_-EG_4_ (**a**) and amplification of the –SH position (**b**).

Figure 1a presents the FTIR spectra of POSS-SH_8_ and POSS-SH_4_-EG_4_, highlighting structural modifications after the partial substitution of –SH groups with PEGDMA. The introduction of PEGDMA, which contains carbonyl (C=O) functional groups, resulted in the appearance of a strong stretching vibration peak at 1736 cm^−1^, confirming the presence of ester bonds. As shown in Figure 1b, the characteristic stretching vibration peak of the thiol (–SH) bond at 2550 cm^−1^ exhibited a gradual decrease in intensity, indicating the reaction of –SH groups during the grafting process. Notably, as the PEGDMA content increased, the attenuation of the –SH peak became more pronounced, suggesting progressive thiol consumption. However, the –SH peak did not completely disappear, demonstrating that a portion of the thiol groups remained unreacted. These spectral changes provide strong evidence that PEGDMA was successfully grafted onto the POSS framework via a thiol-ene click reaction while preserving a certain amount of residual –SH groups.

**Figure 2:**
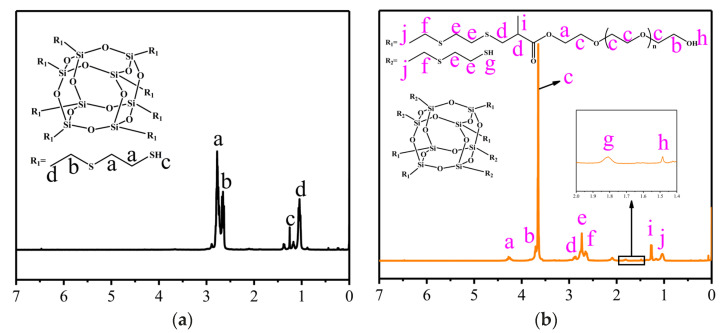
1H NMR spectroscopy of POSS-SH_8_ (**a**) and POSS-SH_4_-EG_4_ (**b**).

Compared to Figure 2a of the NMR spectrum of POSS-SH_8_, characteristic signals at a (4.16 ppm), b (3.6 ppm), c (3.5 ppm), d (2.86 ppm), I (1.37 ppm), and h(1.72 ppm) in Figure 2b of POSS-SH_4_-EG_4_ are generated, respectively. They are corresponding to resonant signals of hydrogen atoms on O=C–O–CH_2_–, –CH_2_–OH, –(CH_2_–CH_2_–O)n–, CH_3_–CH–C=O, CH_3_–, and –OH. The integration area ratio of signals in Figure 2b at j and i is 2.95:4.00, which is close to the theoretical ratio of 3:4. It is consistent with the target product. The results showed that 4 of the 8 sulfydryl groups on POSS-SH_8_ participated in the reaction, and 4 of the sulfydryl groups did not react. Therefore, POSS-SH_4_-EG_4_ was successfully synthesized.

### 3.2. Hydrophilic Evaluation of Coating

**Figure 3:**
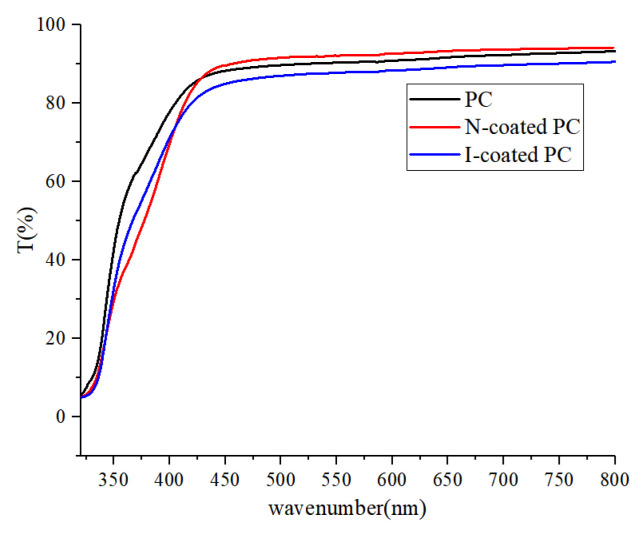
Transmittance contrast of the uncoated blank PC, N-coated PC, and I-coated PC.

The difference in transmittance of the three samples was tested by light transmittance. As shown in Figure 3, the average penetration rate of the uncoated blank polycarbonate (PC) was 89.0%, while that of the N-coated PC was 90.2% and the I-coated PC was 85.9%. The reason for the increased transmittance of N-coated PC was that the PEGDMA coating covers the particles and crack defects on the substrate, and the surface becomes smoother and smoother. The reduced light transmittance of the I-coated PC was due to the certain aggregation of modified POSS in the coating, resulting in side effects such as scattering of light. Even so, its light transmittance was more than 85%, and the transparency was still perfect.

### 3.3. Hydrophilic Evaluation

**Figure 4:**
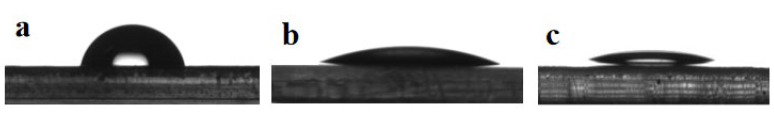
The WCA images of the uncoated blank PC (**a**), N-coated PC (**b**), and I-coated PC (**c**).

The wettability of the coated polycarbonate (PC) substrate was evaluated through water contact angle (WCA) measurements, as illustrated in Figure 4. The WCA of the uncoated blank PC was 82.5°, and the N-coated PC surface was 8.36° ± 2.31°. Because the surface of the N-coated PC was easy to be destroyed by the water drop infiltration and became white, here it was mainly explained for the I-coated PC. The WCA test provided a quantitative measure of the surface’s hydrophilicity, with lower contact angles indicating stronger water affinity. From the camera-captured images. It was evident that the deposited water droplets on the coated surface spread out extensively, forming a thin film-like layer rather than maintaining a spherical shape. This behavior suggested that the coating significantly enhances surface hydrophilicity, facilitating rapid water dispersion. The measured WCA value of the I-coated PC was 8.36° ± 2.31°, confirming its superhydrophilicity. This extremely low contact angle suggested that the surface could effectively prevent water droplet formation, which was crucial for applications requiring antifogging, antifrost, and self-cleaning properties. The small standard deviation (±2.31°) further demonstrated the coating’s uniformity and consistent wettability across the surface.

### 3.4. Antifogging Analysis

**Figure 5:**
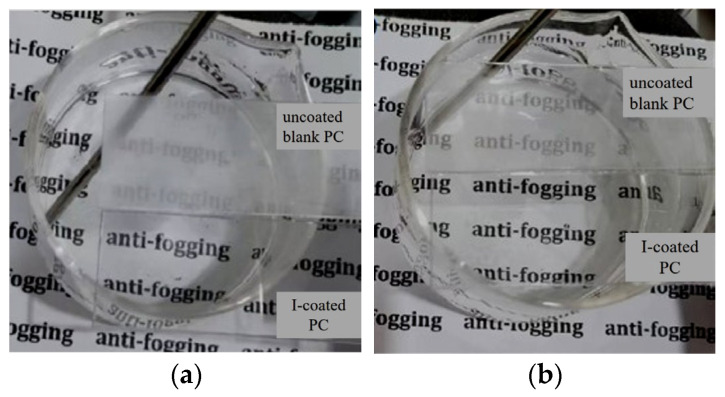
Top-view photographic images of the samples taken after exposing them to the hot vapor ((**a**), 100 °C and (**b**), 50 °C) for 30 s (upper: the uncoated blank PC; lower: I-coated PC).

To assess and compare the antifogging performance of the hydrophilic coating on polycarbonate (PC), both the uncoated blank PC and the I-coated PC were placed above the same beaker filled with hot water. A piece of paper with printed black text was positioned beneath the beaker to observe any changes in transparency. The I-coated PC was oriented with the coating side facing the hot water, while the uncoated blank PC served as a control. Figure 5 illustrates the fog formation on both the uncoated blank PC and the I-coated PC under hot-fog vapor conditions. After 15 s of exposure to the hot water vapor, it was evident that the I-coated PC maintained its high transparency, regardless of the temperature of the hot water (whether at 50 °C or 100 °C). The text on the paper beneath the beaker remained clearly visible through the I-coated PC, whereas the text was indistinct when viewed through the uncoated blank PC. Interestingly, for the I-coated PC, the hot fog vapor condensed into a continuous water film, which allowed light to pass through the sample without significant scattering. This continuous water layer facilitated the transmission of light, thereby preventing fogging and maintaining optical clarity. On the other hand, under identical conditions, the uncoated blank PC fogged up rapidly. Small water droplets condensed on its surface, scattering the incident light and significantly degrading transparency. This comparison highlights the exceptional antifogging capabilities of the hydrophilic coating, achieved through its superior hydrophilic effect. The water droplets on the coated surface spread out quickly, preventing the formation of fog and maintaining transparency. Unlike most conventional organic hydrophilic coatings, the superhydrophilic coating modified with hydrophilic POSS showed no signs of significant whitening, cracking, or peeling after being exposed to hot water at 100 °C for 10 min. This excellent durability suggested that POSS played a crucial role in enhancing the strength and water resistance of the coating, making it highly resilient under harsh conditions.

### 3.5. Antifrost Test

**Figure 6:**
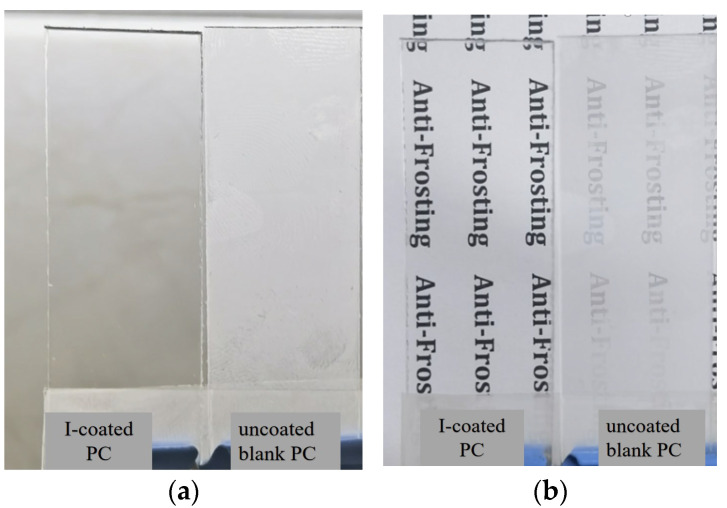
Upward-view and top-view photographic images of the samples were taken after exposing them to the hot vapor ((**a**), 100 °C and (**b**), 50 °C) for 30 s (right: the uncoated blank PC; left: I-coated PC).

The antifrost performance was evaluated by simulating a transition from a low-temperature environment to a room-temperature setting, to mimic real-world conditions where condensation may occur. Both the I-coated PC and the uncoated blank PC were placed in a refrigerator at −5 °C for 15 min. After this period, the samples were then exposed to ambient air at 25 °C and 60% relative humidity, and the condensation behavior on the surface of the coatings was immediately recorded. In the antifrost test, the I-coated PC removed from the cold environment remained clear and free from condensation, whereas the uncoated blank PC fogged up and became opaque within a short period, as shown in Figure 6a. To visually compare the antifrost performance, a piece of paper with black text was placed beneath the samples, as depicted in Figure 6b. From the top-view images, it was evident that the I-coated PC remained transparent, with the black text clearly visible through the sample. In contrast, the uncoated blank PC appeared opaque, and the black text beneath was indistinct, confirming that the coating successfully prevented the formation of frost and maintained optical clarity.

### 3.6. Self-Cleaning Performance

**Figure 7:**
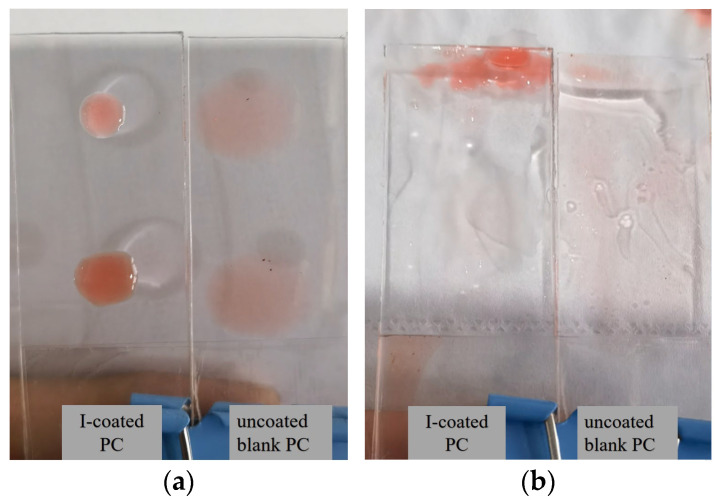
Photographs of the simulated pollutant states on surfaces of the I-coated PC (left) and the uncoated blank PC (right) which were placed horizontally (**a**) and placed by an angle (30°) of inclination (**b**).

In this test, 2 cm × 6 cm rectangular splines were cut, 0.1 mL of oil contaminants were dropped on the surface, and then three drops of water were dropped from the top end of the oil drop, the top distance from the oil drop was about 1 cm, and then tilted down 30 angles to test the status of the oil drop. As shown in Figure 7, the peanut oil had no spread on the surface of the I-coated PC but expanded on the uncoated blank PC surface (Figure 7a). After adding water droplets and tilting by 30 angles, the oil droplets on the I-coated PC appeared floating on the surface of the water film, and quickly slid down to the bottom of the spline within 2S, without any oil droplets remaining on the surface. The oil droplets on the surface of the PC substrate only continued to spread around under the gravity action of the water droplets and, basically, covered the entire PC surface. This indicated that the I-coated PC surface had excellent hydrophilic properties and that the water droplets were able to quickly spread on its surface into a water membrane, which floated the less dense oil droplets. This performance suggested the I-coated PC surfaces have good self-cleaning characteristics for underwater erosion.

### 3.7. Water Resistance

**Figure 8:**
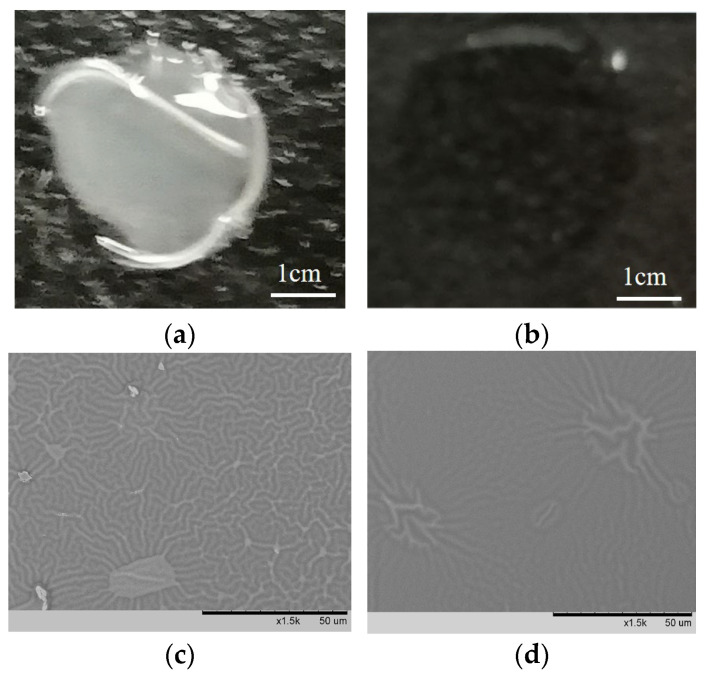
The surface water droplet condition ((**a**) N-coated PC, (**b**) I-coated PC) and SEM of N-coat and I-coat after 96 h of water resistance test ((**c**) N-coated PC, (**d**) I-coated PC).

To evaluate the water resistance and durability of the coating surface, the I-coated PC and N-coated PC were tested. Both samples were subjected to accelerated testing in a constant temperature and humidity chamber at 85% humidity and 85 °C for 96 h to simulate prolonged exposure to harsh environmental conditions. Following this, the samples were dried in an oven at 60 °C for 20 min. The surface contact angle and any apparent changes in the coatings were then analyzed, and the results are shown in Figure 8. As shown in Figure 8a, the surface of the N-coated PC was partially cracked and peeled off, indicating poor mechanical strength and weak adhesion to the substrate. This suggests that in the absence of hydrophilic modified POSS, the coating lacks the necessary structural integrity to withstand high humidity and temperature conditions. As a result, water droplets easily spread on the surface, leading to penetration within the layer, which compromised the overall integrity of the coating. In contrast, the I-coated PC demonstrated superior water resistance and durability. The contact angle of the I-coating remained relatively stable, ranging from approximately 8.5° to 7.3°, indicating that the coating retained its hydrophilic properties even after prolonged exposure to harsh conditions. As shown in Figure 8b, there were no significant signs of whitening or wrinkling, and no visible changes were observed on the surface, showcasing the coating’s stability under high temperature and humidity. This indicates that the addition of hydrophilic modified POSS significantly improved the mechanical strength and water resistance of the coating.

The SEM images further confirmed the differences between the two surfaces. As shown in Figure 8c, parts of the N-coated PC surface that did not peel off still exhibited large-scale cracks and extensive wrinkling after the durability test, revealing that the coating’s structure was severely compromised. In contrast, the I-coated PC surface in Figure 8d showed only minor wrinkling in a few areas, with no significant cracking or leakage from the underlying substrate. The lack of major structural damage in the I-coated PC surface indicated that the POSS-modified coating maintained its integrity even under harsh conditions. The presence of wrinkles in the N-coated surface also contributed to the observed decrease in the water contact angle, as the rough surface texture promoted the spreading of water droplets. In contrast, the I-coated PC maintained a more stable and hydrophilic surface with the stabilizing effect of the POSS modification. In summary, the results clearly demonstrated that the addition of hydrophilic modified POSS greatly enhances the water resistance and durability of the coating. The improved mechanical strength and resistance to environmental stressors suggested that POSS-modified coating was more suitable for applications requiring long-term performance in challenging conditions.

### 3.8. Surface Structure

**Figure 9:**
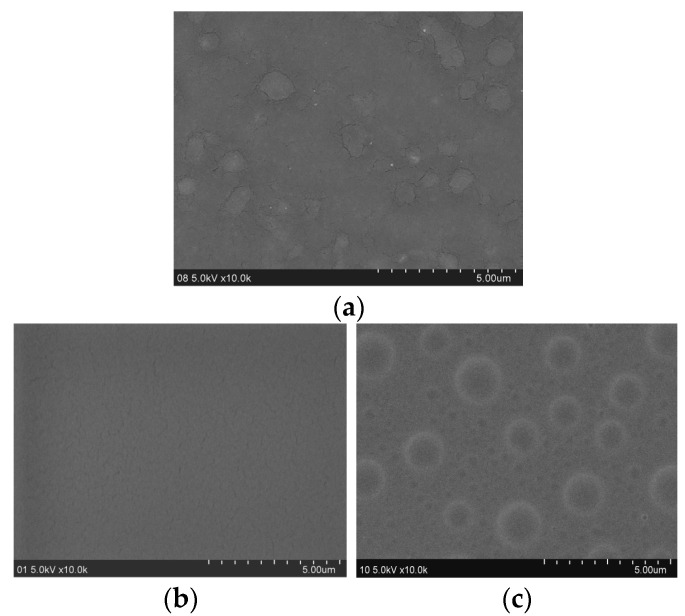
SEM diagram of the uncoated blank PC (**a**), N-coated PC surface (**b**), and I-coated PC surface (**c**).

SEM of PC substrate, N-coated PC, and I-coated PC were characterized to clear the effect of hydrophilic modified POSS on the I-coated PC. In Figure 9a, mall particles and cracks not completely melted appeared on the seemingly transparent and smooth PC substrate. The small defects on the surface of the PC substrate were covered by the N-coated PC, but the smaller cracks appeared in the N-coated PC (Figure 9b). There cracks were caused by the subsequent release of the internal stress existing in the surface shrinkage during UV curing of the coating. With the addition of hydrophilic modified POSS, a circular island-like structure appeared in the I-coated PC, containing not only about 300 nm, but also 2–3 micron clusters, and the boundaries between the clusters and the surroundings were blurred (Figure 9c). These isolated islands were caused by the migration, aggregation, and microphase separation of hydrophilic modified POSS to the surface. On the one hand, the rough surface structure formed by these islands could effectively promote the rapid spreading of water droplets on the coating surface to form a film, thereby improving the hydrophilicity of the I-coated PC surface. On the other hand, the inorganic POSS core inside the island could effectively prevent moisture from diffusing into the coating and improve the water resistance of the coating.

### 3.9. Surface Element

**Figure 10:**
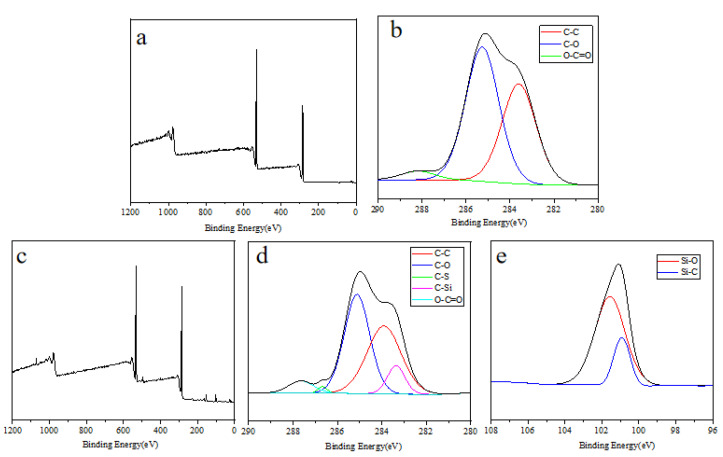
C 1s core-level XPS spectra of N-coating (**a**,**b**); I-coating (**c**,**d**); Si 2p core-level XPS spectra of I-coating (**e**).

The surface full-spectrum XPS results of the N-coating were shown in Figure 10a, where the peaks appearing at the binding energies of 532.3 eV and 285.2 eV were O 1s and C 1s, respectively. The Figure 10b showed the XPS pattern of the high-resolution C 1s peak. According to the coating composition, C 1s were divided into three Gaussian fitting peaks: O=C–O (288.3 eV), C–O (285.2 eV), and C–C (283.6 eV). A higher proportion of C–O bonds indicated a high content of polyether segments (–CH_2_–O–) on the surface of the coating, and –CH_2_–O– imparted hydrophilicity to the coating effectively. The surface full-spectrum XPS results of the POSS-containing I-coating are shown in Figure 10c. The peaks appearing at binding energies of 531.7, 284.8, 162.2, and 101.5 eV were O 1s, C 1s, S 2p, and Si 2p, respectively. Figure 10d is a high-resolution C 1s peak division XPS diagram. According to the coating composition, C 1s can be divided into O=C–O (287.5 eV), C–S (285.6 eV), C–O (285.1 eV), C–C (283.4 eV), and C–Si (284.2 eV) five Gaussian fitting peaks. The ratio of the C–O peak area to the C–C peak area is about 1.1: 1. Figure 10e is a high-resolution Si 2p peak division XPS diagram. According to the molecular structure of POSS, Si 2p was divided into two Gaussian fitting peaks: Si–O (101.5 eV) and Si–C (100.9 eV). The area of the Si–O peak was significantly larger than that of the Si–C bond, and this indicated that the inorganic core of POSS migrated from the inside of the coating to the surface.

## 4. Conclusions

This study aimed to develop a hydrophilic coating, utilizing the synthetic building block POSS-PEGMA. By incorporating PEGMA into the modified POSS structure, a synergistic effect was achieved, resulting in improved performance in antifogging, antifrost, and self-cleaning properties. The inclusion of POSS significantly enhanced the mechanical strength and water resistance of the coating. The surface roughness observed in the coating was attributed to the migration, aggregation, and microphase separation of the hydrophilic modified POSS, which facilitated the rapid spreading of water droplets, forming a uniform film that improved the hydrophilicity of the I-coated PC surface. Additionally, the inorganic POSS core present in the island-like structures effectively prevented moisture diffusion into the coating, further enhancing its water resistance. The coating was produced using a UV-curable method, which is commonly employed in large-scale production, offering an efficient approach for manufacturing coatings in industrial applications.

## Data Availability

The original contributions presented in this study are included in the article/Supplementary Materials. Further inquiries can be directed to the corresponding author.

## Supplementary Materials

The following supporting information can be downloaded at https://www.mdpi.com/article/10.3390/polym17050599/s1. Supplementary File S1: Comparative analysis of our coating with similar studies.
