# Supplementary material for: Study of Polyhedral Oligomeric Silsesquioxane-Modified Superhydrophilic Transparent Coating in Antifogging, Antifrost and Self-Cleaning
Source: Polymers (Basel). 2025 Feb 24;17(5):599. doi: 10.3390/polym17050599 (PMC11902433; doi:10.3390/polym17050599)
Supplement: Supplementary file 1 [file polymers-17-00599-s001.zip › polymers-3445799-supplementary.pdf]

Supplementary File 1

Comparative analysis of our coating with the similar studies

Reference

1 Yang Y, Zeng L, Li X, et al. Hydrophilic/hydrophobic poly (AA-co-BA-co-BPA) anti-fog coating with excellent water resistance and self-healing properties[J].*Progress in Organic Coatings*, 2024, *187*,108071.
2 Manabe K , Norikane Y .Graphene composite self-healing antifog/frost-resist transparent coatings with zwitter-wettability[J].*Surfaces and Interfaces*, 2023, *42*,103363.
3 Zhong H, Liu X, Yu B, Zhou S. Fast UV-Curable Zwitter-Wettable Coatings with Reliable Antifogging/Frost-Resisting Performances. *Biomimetics*[J]. 2022, *7(4)*,162.
4 England M W, Urata C, Dunderdale G J, et al. Anti-Fogging/Self-Healing Properties of Clay-Containing Transparent Nanocomposite Thin Films[J]. *ACS Applied Materials & Interfaces*, 2016, *8(7)*,4318-4322.
5 Ma Z, Liu Y, Feng K, et al. "Brush-like" Amphiphilic Polymer for Environmental Adaptive Coating[J]. *ACS applied materials & interfaces*, 2022, *14(16)*,18901-18909.
6 Ren, JL, Kong RX, Gao YJ, Zhang LB, Zhu JT. Bioinspired adhesive coatings frompolyethylenimine and tannic acid complexes exhibiting antifogging, self-cleaning, and antibacterial capabilities[J]. Journal of Colloidand Interface Science, 2021, *603,* 406-414.

Study No.	Brief introduction	Antifogging property	Antifrost property	Comparison
1	The study reports a multifunctional coating with anti-fog and anti-frost properties using butyl acrylate (BA), acrylic acid (AA) and 4-benzoylphenyl acrylate (BPA) as the polymer monomers.	The antifogging tests were carried out by placing the coated samples above 5 cm over the hot water (80 ℃) for 10 s.The coated samples exhibited antifogging behaviors.	The coated samples were stored in a refrigerator at -20 ℃ for 1 h and then exposed to the ambient condition (-20℃, 35%~40% relative humidity) showed an frost-resisting behavior	1,The substrate in the study is the plasma-treated glass plate, hence the coating of this study is potential for application in construction and shipbuilding<br>2,The temperature of antifogging test is carried out at 80 ℃ for 10 s while our study is carried out at 100 ℃ for 15 s. More stringent testing conditions seems suggested better antifogging property.
2	The study reports a polymer multilayer graphene composite coatings exhibit simultaneous antifogging and self-healing properties via layer-by-layer self-assembly without any post-treatment.	The coatings used in this study had zwitter-wettability properties with water droplet contact angles well above 40◦ , making them ineffective in preventing fog formation under conventional conditions.	The antifrost performance was investigated on the glass substrate refrigerator to -18 ℃ for 1 h and then exposed to a high-humidity environment (80% relative humidity) at 25 ℃ the coated areas maintained high transparency throughout the test.	1,The surface wettability of the fabricated coatings was generally nonhydrophilics, which was attributed to the hydrophobic nature of graphene<br>2,The black color and decreased transparency of coating influenced by black graphene content limit its applications.
3	The study reports a zwitter-wettable antifogging and frost-resisting coating through a fast UV-curable cross-linking of copolymer with benzophenone	The antifogging tests were carried out by placing the coated samples above 5 cm over the hot water (80 ℃) for 60 s.The coated samples	The coated samples were stored in a refrigerator at -20 ℃ for 2 h and then exposed to the ambient condition (-20 ℃, 35%~40% relative humidity)	The strategy of this study to achieve antifogging and antifrost properties is different from our study. In our study,a highly hydrophilic coating with a water contact angle below 10° was developed.<br>The coated water droplets in this study required a

	groups.	exhibited antifogging behaviors.	showed an obviously enhanced frost-resisting behavior.	long response time to spread a film, which could not achieve rapid antifogging performance.
4	The study reports an antifogging films, including glass slides, silicon, copper and PMMA, by spin-coating a mixture of polyvinylpyrrolidone and aminopropyl-functionalized, nanoscale clay platelets.	The resulting films were superhydrophilic and showed more than 90% transmission of visible light, as well as excellent antifogging and self-healing properties. The anti-fogging performance still has excellent performance after 20 cycles.	The article did not study the coating's antifrost property.	The coating system was simple and reproducible, with low cost; the composite membrane can gradually self-heal. However, if the coating with this excellent performance was obtained, it needed 100℃ to bake for 3 hours, and the thermosensitive transparent plastic was easy to be seriously deformed or damaged during the curing process.
5	The study reports an environment-adaptive multifuncti onal coating based on the copolymer via the UV-curing method synthesized by a brush-like amphiphilic copolymer of poly methacryl- oxyethyl dimethyl butyl ammonium bromide poly- dimethylsiloxane (pMDBAB-PDMS).	The hygroscopic properties of the PMDBAB units embedded in the coating mesh gives it good self-cleaning, antifogging, antistatic, ultra-low friction coefficient and transparent property.	This study did not describe its excellence antifrost property.	The transition in dry and wet environments requires some response time. If there is a rapid temperature difference change during this period, the fog is difficult to remove, which affects related applications.
6	The study reports a simple yet robust method to fabricate a bioinspired adhesive coating based on	The hydrophilicity of the coated substrates can be significantly enhanced with their water contact angle less	The samples before and after PEI-TA coating were firstly stored in a refrigerator (-20 ℃) for 30 min. Digital photographs	This coating was dissolved in DI water with a concentration of 40 mg/mL, so it should take a long time to volatilize the water. Moreover, the process requires HCl to adjust the PH value, which

	polyethyleneimine (PEI) and tannic acid (TA) complexes, exhibiting excellent antifogging, self-cleaning,<br>and antibacterial properties.	than 10.coated safety goggles display excellent durability and antifogging capability compared to the commercial antifogging safety goggles and commercial antifogging agents coated safety goggles under 65 ℃ vapor condition for 2 h.	were taken immediately for the samples at ambient conditions. The PEI-TA coated glass slide substrate still exhibited high transmittance over (88.5%) than the bare substrate (37.5%).	increases the risk of operation.
My Study	We introduced the modified hydrophilic POSS into the hydrophilic mixture, coated it on the PC substrate, and cured the mixture by UV radiation to obtain the transparent coating.	Because the water drop Angle is below 10, there is no fog at high temperature (100℃) or low temperature (50℃).	After the low temperature (-5℃) environment changes to room temperature, the surface of the coated PC plate is formed from any frost or fog.	The addition of POSS solves the problem of poor water resistance of the hydrophilic coating, and the nano-modified POSS has little influence on the transparency of the coating. This method is suitable for large area rapid coating and curing, and suitable thermal substrate.
